# Compositional Dependence of Curie Temperature and Magnetic Entropy Change in the Amorphous Tb–Co Ribbons

**DOI:** 10.3390/ma14041002

**Published:** 2021-02-20

**Authors:** Xin Wang, Ding Ding, Li Cui, Lei Xia

**Affiliations:** 1 College of Engineering, Shanghai Polytechnic University, Shanghai 201209, China; wangxin2020@126.com (X.W.); cuili@sspu.edu.cn (L.C.); 2 Institute of Materials, Shanghai University, Shanghai 200072, China; 3 Center for Advanced Microanalysis, Shanghai University, Shanghai 200444, China

**Keywords:** amorphous materials, curie temperature, magnetic entropy change

## Abstract

The Curie temperature (*T_c_*) and magnetic entropy change (−Δ*S_m_*), and their relationship to the alloy composition of Tb–Co metallic glasses, were studied systematically in this paper. It was found that, in contrast to the situation in amorphous Gd–Co ribbons, the dependence of Tc on Tb content and the maximum −Δ*S_m_* vs. *T_c_* -2/3 plots in Tb–Co binary amorphous alloys do not follow a linear relationship, both of which are supposed to be closely related to the non-linear compositional dependence of Tb–Co interaction due to the existence of orbital momentum in Tb.

## 1. Introduction

In recent years, with the increasing demands for reducing energy consumption and the mitigating global warming, new refrigeration technologies have been intensively investigated. Amongst these innovative refrigeration technologies, magnetic refrigeration (MR) technology has been extensively concerned since it is free contributing to the depletion of the ozone layer and due to its long-service life, superior to the that of traditional vapor compression refrigeration technology. Therefore, experts in the related research fields are paying more and more attention to the development of novel magnetocaloric materials in the last two decades because the efficiency of the magnetic refrigerator is determined by the magnetocaloric properties of its magnetic refrigerant [1,2,3,4,5].

Amorphous magnetocaloric alloys, as an important category of magnetic refrigerant, show potential applications from the perspective of magnetic refrigerators because they possess a rather broad “hillside” of magnetic entropy change (−Δ*S_m_*) and a low but adequate maximum −Δ*S_m_* (−Δ*S_m_^peak^*), which results in an ultrahigh refrigeration capacity (*RC*) [5,6,7,8,9,10,11,12,13,14,15,16,17,18,19,20,21,22,23,24,25,26,27]. This makes it possible to design and manufacture metallic glass composites with a flattened −Δ*S_m_* peak within a tailorable temperature span because magnetic refrigerant with a flattened −Δ*S_m_* peak within the cold end and the hot end of a refrigerator is expected to be optimal in an Ericsson cycle [6,7,8,9].

In the preliminary works, metallic glasses consisting of rare earth (RE) elements and transition metals (TM) elements, especially the Gd–TM-based amorphous alloys, have demonstrated excellent magnetocaloric properties [8,9,10,11,12,13,14,15,16,17,18,19]. However, RE–TM-based amorphous materials containing other rare earth (Tb, Dy, etc.) metals usually exhibit spin-glass-like behaviors and high coercivity at low temperature [19,20,21,22,23,24,25,26,27]. The hard magnetic properties make the magnetocaloric behaviors irreversible and thus deteriorate the application perspective of these metallic glasses. Therefore, it is necessary to systematically observe the magnetic and the magnetocaloric properties of the Tb/Dy–TM binary metallic glasses, which may be helpful to understand the relationship between alloy composition and the magnetic properties, the origin of coercivity and their influences on the magnetocaloric effect of these metallic glasses.

In this paper, the magnetic properties and magnetocaloric effect, including the Curie temperature (*T_c_*), coercivity, spin freezing temperature (*T_f_*) and the −Δ*S_m_* of the binary Tb–Co amorphous alloys were measured. Based on these results, the dependence of Curie temperature on the composition of the alloys and the relationship between the maximum −Δ*S_m_* and the Curie temperature of the glassy samples were established, and the mechanism involved was investigated.

## 2. Materials and Methods

Sample synthesis: Tb and Co pure metals with a purity higher than 99.9 at.% were mixed together according to the stoichiometric compositions of Tb_x_Co_100−x_ (x = 45, 50, 55, 60, 62.5) and were arc-melted, respectively, into the shape of ingots in the presence of a Ti getter under an Ar atmosphere. Ribbons of each alloy were manufactured by means of melt-spinning method under the protection of an argon atmosphere. The thickness of the ribbons is ~40 μm in average.

Structural characterization: The disordered structure of the Tb_x_Co_100−x_ ribbons was confirmed by a Rigaku X-ray diffractometer (model D/max-rC) with Cu *K_α_* source.

Magnetic measurements: the *T_c_* and *T_f_* of the ribbons were obtained from the magnetization vs. temperature (*M*–*T*) curves measured under a magnetic field of 0.03 T (Tesla) after a cooling under a zero field (ZFC) and a cooling under a magnetic field (FC) of 0.03 T. Saturation magnetization (*M_s_*) and the coercivity of the ribbons were obtained from the hysteresis loops measured under a field of 5 T. The plots of −Δ*S_m_* vs. temperature ((−Δ*S_m_*)-*T* curves) were constructed according to the Maxwell equation from the isothermal magnetization (*M*–*H*) curves measured at various temperatures under 5 T. The *M*–*T* curves, hysteresis loops and *M*–*H* curves were measured on a vibrating sample magnetometer module of a quantum design PPMS Evercool II system. Measurement precision is less than 5 × 10^−6^ emu/T(Tesla).

## 3. Results

**Figure 1:**
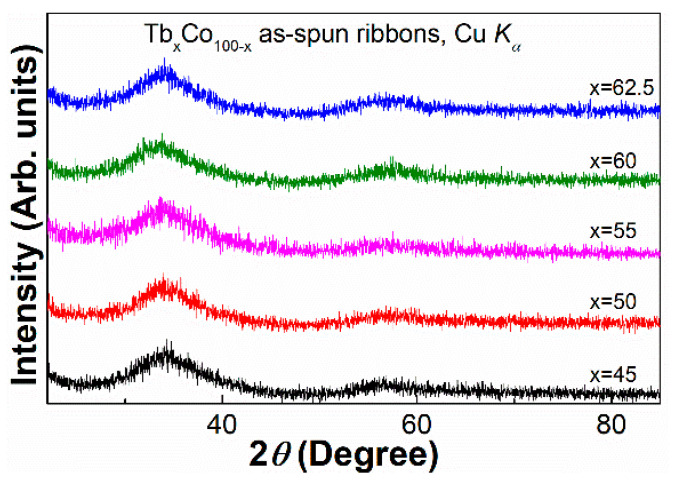
XRD patterns of the Tb_x_Co_100−x_ (x = 45, 50, 55, 60, 62.5) as-spun ribbons.

Figure 1 illustrates the X-ray diffraction (XRD) patterns of the as-spun Tb_x_Co_100-x_ (x = 45, 50, 55, 60, 62.5) ribbons. Only broad humps representing the first diffuse halo and the absence of visible crystalline peaks indicate the formation of a fully amorphous phase in each ribbon.

**Figure 2:**
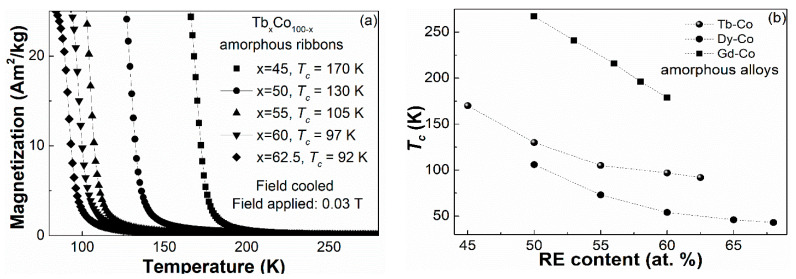
(**a**) FC *M–T* curves of the Tb_x_Co_100-x_ (x = 45, 50, 55, 60, 62.5) amorphous ribbons; (**b**) the relationship between the *T_c_* and the RE element content in the Tb_x_Co_100-x_, Dy_x_Co_100-x_ and Gd_x_Co_100-x_ amorphous alloys.

Figure 2a displays the FC *M*–*T* curves of the Tb_x_Co_100−x_ (x = 45, 50, 55, 60, 62.5) glassy samples measured under a field of 0.03 T. By taking the derivation of the *M*–*T* curves, we can obtain the Curie temperature of the glassy samples as follows: 170 K for Tb_45_Co_55_, 130 K for Tb_50_Co_50_, 105 K for Tb_55_Co_45_, 97 K for Tb_60_Co_40_ and 92 K for Tb_62.5_Co_37.5_ [28], as summarized in Table 1. The variation of the Curie temperature with the composition of the Tb_x_Co_100−x_ glassy samples, and the Dy_x_Co_100−x_ as well as Gd_x_Co_100−x_ binary metallic glasses for comparison purpose [11,23], are plotted, respectively, in Figure 2b. Along with the increase in RE content, the Curie temperature of the three kinds of RE–Co binary metallic glasses decreases monotonously. However, one can find that the compositional dependence of *T_c_* follows a linear relationship in Gd–Co binary alloys, which in contrast, is non-linear in Tb–Co and Dy–Co binary alloys.

In order to reveal the mechanism for the dependence of *T_c_* on composition in these RE–Co binary metallic glasses, it is necessary to study the interactions between atoms in these metallic glasses, including the Co–Co (direct) interaction, the RE–RE (indirect) interaction and RE–Co (indirect) interaction. If the variation of Curie temperature with the composition of the RE–Co binary glassy alloys is only induced by the RE–RE interaction, the relationship between the Curie temperature and the RE content in the RE–Co binary metallic glasses agrees well with the RKKY (Ruderman-Kittel-Kasuya-Yosida) indirect interaction model, that is, *T_c_* will have a linear relationship with the G factor, which is a physical quantity simply related to the mole fraction of the RE element in the alloy system that contains merely one type of RE atoms [17,26]. For amorphous alloys containing only one RE element, the G factor is proportional to the molar fraction of the RE atoms. Therefore, from the viewpoint of 4f–4f indirect interaction, the compositional dependence of Tc in Tb–Co amorphous alloys should be linear. However, in addition to the 4f–4f indirect interaction between the RE–RE atoms, there are two other kinds of interactions in RE–TM-based amorphous alloys: 3d–3d direct interaction between the TM–TM atoms and the 3d–4f indirect interaction between the RE–TM atoms. The influence of the 3d–3d direct interaction on the Curie temperature in the RE–TM-based amorphous alloys is supposed to be similar to that of the 4f–4f indirect interaction. This is understandable because there is only (100%) 3d–3d direct interaction in a pure TM metal, and no (0%) 3d–3d direct interaction in alloys free of TM elements. The contribution of the 3d–3d direct interaction is proportional to the molar fraction of the Co element in Tb–Co amorphous alloys and the compositional dependence of Tc in Tb–Co amorphous alloys is still linear from the viewpoint of 3d–3d direct interaction. However, the effect of 3d–4f indirect interaction on the Curie temperature in RE–TM-based amorphous alloys is more complicated [23]. On the other hand, the direct interaction between Co atoms is largely influenced by the surrounding environment of a Co atom, which means that the addition of the large-size RE atoms (e.g., Gd, Tb, Dy...) give rise to the expansion of the distance between Co atoms and thus weaken the interactions between Co atoms. In other words, if the variation of Curie temperature with the composition of the RE–Co binary glassy alloys is considered to be only induced by the Co–Co direct interaction, the Curie temperature of the RE–Co binary metallic glasses will decrease with the decreasing content of Co. The dependence of *T_c_* on the Co–Co interaction probably resembles the case of the RE–RE interaction, which indicates the linear relationship between *T_c_* and Co content when only Co–Co interaction is considered. The assumption can be ascertained in the binary Gd–Co metallic glasses [11]. Unlike other RE metals such as Tb and Dy, Gd has no orbital momentum due to its stable half-full 4*f* shell, and thus the interaction among Gd–Co atoms can generally be ignored. As such, the Curie temperature of the Gd–Co metallic glass system ultimately depends on the co-effect of the Co–Co direct interaction and Gd–Gd indirect interaction. Considering that the *T_c_* exhibits a linear relationship with the composition in the Gd–Co metallic glasses, as shown in Figure 2b, and the concentration of Gd element and *T_c_* are linearly related according to the RKKY model, the relationship between Co concentration and *T_c_* induced by the Co–Co interaction should also be linear. It is reported that the *T_c_* variation induced by the Gd–Gd indirect interaction is nearly equal to the variation of the *T_c_* induced by the direct interaction between Ni–Ni atoms, which makes the Curie temperature remains constant near 123 K in the Gd–Ni binary metallic glasses [12]. In contrast, the increase in Gd content in Gd–Co metallic glasses, that is, the decrease in Co concentration, makes the Curie temperature decrease linearly from 267 to 179 K because the direct interaction between Co atoms is much stronger than that of the Ni atoms.

The compositional dependence of the Curie temperature in the binary Tb–Co and Dy–Co amorphous alloy systems; however, is more complicated because of the existence of RE–Co interactions [23]. For the binary Tb–Co metallic glasses in the present work, the Tb–Co interaction is zero either in pure Tb or in pure Co, but exists in Tb–Co compounds, changing with the composition of the alloy and reaching a maximum value at a certain composition. As a result, the relationship between *T_c_* and Tb content in the Tb–Co metallic glass system will not be the linear relationship in Gd–Co alloys. The parabolic-like nonlinear curve exhibited in Tb–Co binary amorphous alloys is somewhat similar to the one in Dy–Co binary alloys, indicating the similar variation trend of *T_c_* induced by the RE–Co interaction in the binary Dy–Co and Tb–Co amorphous alloys.

**Figure 3:**
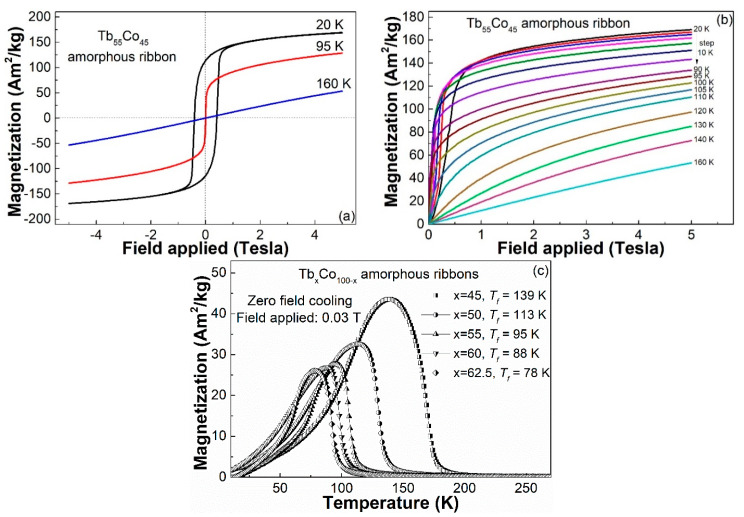
(**a**) The hysteresis loops and (**b**) the isothermal *M*–*H* curves of the Tb_55_Co_45_ amorphous ribbon measured at different temperature under a magnetic field of 5 T; (**c**) zero field (ZFC) *M–T* curves of the Tb_x_Co_100−x_ (x = 45, 50, 55, 60, 62.5) amorphous ribbons.

In our preliminary work, the Gd–Co amorphous alloys are soft magnetic with almost zero hysteresis because the stable half full electron arrangement in the 4*f* shell of Gd atom does not produce orbital momentum and thus Gd has a relatively small magneto-crystalline anisotropy. In contrast, the unstable electronic arrangement of the 4*f* shell in the Tb or Dy atom gives rise to the existence of orbital momentum and produces a large magneto-crystalline anisotropy, which is expected to result in a relatively high coercivity at low temperature in Tb–Co and Dy–Co amorphous alloys. As shown in Figure 3a, the Tb_55_Co_45_ amorphous ribbon is hard magnetic with a coercivity of ~326 kA/m at 20 K, soft magnetic at 95 K and paramagnetic at 160 K. The high coercivity at 20 K is due to the random magnetic anisotropy (RAM) which exists in Tb-based amorphous system [20,21,22], which will lead to the preferential orientation between the magnetic moments, destroy the macroscopic effective anisotropy directions of the magnetic order and thereby produce the hysteresis.

In order to reveal the magnetocaloric properties of Tb–Co binary metallic glass and explore the mechanism of the magnetic behaviors involved in a more intensive way, it is necessary to measure the isothermal magnetization curves at various temperatures ranging from a very low temperature of 20 K to a temperature of 160 K above the *T_c_*. To prevent the effect of magnetization history on the *M*–*H* curve at low temperature, the glassy ribbon was heat treated to eliminate residual magnetism during the measurement process. As shown in Figure 3b, the initial magnetization obviously increases with the increasing temperature from 20 to 90 K within a low range of magnetic field before saturation magnetization, which indicates the spin glass behavior of the Tb_55_Co_45_ metallic glasses [19,20,21,22,23,24,25,26,27]. The spin glass behaviors of the amorphous Tb_x_Co_100−x_ (x = 45, 50, 55, 60, 62.5) samples are also illustrated in Figure 3c by their ZFC *M*–*T* curves. The spin freezing temperatures of these samples, as listed in Table 1, can be obtained from their ZFC *M*–*T* curves.

**Figure 4:**
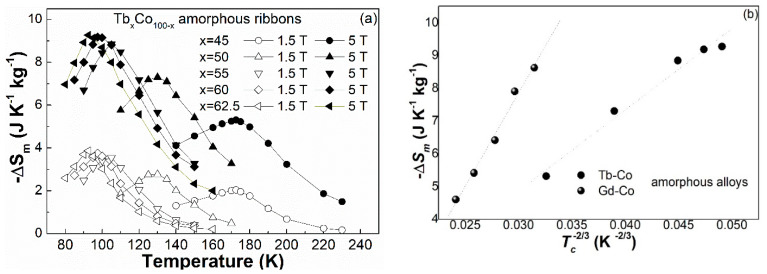
(**a**) The (−Δ*S_m_*)-*T* curves of the Tb_x_Co_100−x_ (x = 45, 50, 55, 60, 62.5) amorphous ribbons under the magnetic field of 1.5 T and 5 T; (**b**) the linear fittings of *T_c_*^−2/3^ vs. −Δ*S_m_^peak^* plots for the Gd–Co and Tb–Co amorphous alloys.

According to the isothermal *M*–*H* curves of the Tb_x_Co_100−x_ (x = 45, 50, 55, 60, 62.5) metallic glasses, the −Δ*S_m_* vs. temperature under various magnetic fields can be obtained. Unfortunately, the spin freezing behavior and the high coercivity at low temperature obviously deteriorate the magnetocaloric properties of these alloys at temperatures below their spin freezing temperature, even decrease the −Δ*S_m_* value to below zero at 20 K. Therefore, we only study the magnetocaloric behaviors of the reversible part above the *T_f_*. The −Δ*S_m_* vs. temperature plots of the Tb_x_Co_100−x_ amorphous samples under the fields of 1.5 T and 5 T are demonstrated in Figure 4a. The −Δ*S_m_^peak^* of the Tb_x_Co_100−x_ metallic glasses increases with the increasing Tb content but decrease with the increasing *T_c_*. The −Δ*S_m_^peak^* values of the Tb_x_Co_100−x_ glassy samples under 1, 1.5, 2, 3, 4 and 5 T are summarized in Table 1. The −Δ*S_m_^peak^* values of the Tb_62.5_Co_37.5_ amorphous alloy are much higher than those of the TbCo amorphous ribbons, which is most likely related to the higher magnetic moment of a Tb_62.5_Co_37.5_ amorphous alloy due to its high Tb concentration and the high magnetic moment of the Tb3+ ions (~9.72 μB) [28]. Considering the potential application perspective the linear −Δ*S_m_^peak^* ∝ *T_c_*^−2/3^ relationship in Gd-based amorphous alloys [10,11,17,29], we constructed the −Δ*S_m_^peak^*-*T_c_*^−2/3^ plots of Tb_x_Co_100-x_ glassy ribbons obtained from the (−Δ*S_m_*)-*T* plots under 5 T, as shown in Figure 4b. Unlike the linear relationship between the −Δ*S_m_^peak^*-*T_c_*^−2/3^ plots in the binary Gd–Co glassy alloys, as also shown in Figure 4b for comparison purposes, the relationship between −Δ*S_m_^peak^* and *T_c_*^−2/3^ in the Tb–Co binary amorphous samples is more like a para-curve than a linear relationship. The non-linear change of the −Δ*S_m_^peak^* and *T_c_*^−2/3^ is also considered to be related to the non-linear relationship between the Tb concentration and Tb–Co interaction in the Tb–Co binary metallic glasses.

## 4. Conclusions

In summary, the compositional dependence of *T_c_* and −Δ*S_m_* of the amorphous Tb_x_Co_100-x_ (x = 45, 50, 55, 60, 62.5) alloys, and the mechanism involved, were investigated in this paper. The magnetic properties and magnetocaloric effect of the binary Tb–Co amorphous alloys were measured systematically. *T_c_* of the Tb–Co metallic glasses were obtained from their FC *M*–*T* curves and the dependence of *T_c_* on the composition of the alloys was constructed. The spin-glass behaviors were observed in the isothermal *M*–*H* curves at low temperature and the ZFC *M*–*T* curves of the Tb–Co glassy alloys. To prevent the deterioration of the magnetocaloric properties by spin freezing behaviors, the (−Δ*S_m_*)-*T* curves of the Tb_x_Co_100-x_ glassy alloys were established above the spin freezing temperature. Based on these results, the −Δ*S_m_^peak^* vs. *T_c_*^−2/3^ plots for the Tb–Co systems were obtained. It can be discovered that the compositional dependence of *T_c_* and the −Δ*S_m_^peak^* vs. *T_c_*^−2/3^ plots in Tb–Co binary metallic glasses does not follow a linear relationship compared with the Gd–Co binary metallic glasses, which is mostly due to the non-linear compositional dependence of Tb–Co interaction due to the existence of orbital momentum in Tb.

## Figures and Tables

**Table 1:** The Curie temperature (*T_c_*), spin freezing temperature (*T_f_*) and −Δ*S_m_^peak^* under various magnetic fields of the Tb_x_Co_100-x_ amorphous ribbons.

Tb_x_Co_100−x_ Ribbons	*T_c_* (K)	*T_f_* (K)	−Δ*S_m_^peak^* (J K^−1^ kg^−1^)						Ref.
			1 T	1.5 T	2 T	3 T	4 T	5 T	
x = 45	170	139	1.45	2.04	2.58	3.56	4.46	5.31	Present work
x = 50	130	113	1.94	2.76	3.52	4.90	6.15	7.30	
x = 55	105	95	2.52	3.54	4.46	6.10	7.54	8.84	
x = 60	97	88	2.67	3.75	4.71	6.39	7.86	9.18	
x = 62.5	92	78	2.77	3.86	4.83	6.51	7.97	9.27	[28]

## Data Availability

The data reported in this article are available on request from the Corresponding author.
